# Identification of Priority Implementation Areas and Configuration Types for Green Infrastructure Based on Ecosystem Service Demands in Metropolitan City

**DOI:** 10.3390/ijerph19138191

**Published:** 2022-07-04

**Authors:** Dongmeng Wang, Yongge Hu, Puxia Tang, Chang Liu, Weihan Kong, Jie Jiao, Krisztina Filepné Kovács, Dezheng Kong, Yakai Lei, Yiping Liu

**Affiliations:** 1 Department of Landscape Architecture, College of Landscape Architecture and Art, Henan Agricultural University, Zhengzhou 450002, China; dongmengwang@stu.henau.edu.cn (D.W.); huyonggec@gmail.com (Y.H.); puxiatang@126.com (P.T.); lunaliu0124@163.com (C.L.); guihuashi_jiaojie@126.com (J.J.); kdzhenau@henau.edu.cn (D.K.); lykfjyl@163.com (Y.L.); 2 Department of Urban and Regional Planning, Faculty of Built Environment and Surveying, Universiti Teknologi Malaysia, UTM Skudai, Johor 81310, Malaysia; kongweihan@graduate.utm.my; 3 China Fortune Land Development Co., Ltd., Tower A, Gateway Plaza, No.18 Xiaguangli, East 3rd Ring North Road, Chaoyang District, Beijing 100027, China; 4 Institute of Landscape Architecture, Urban Planning and Garden Art, Hungarian University of Agricultural and Life Sciences, No. 29–43 Villányi út, 1118 Budapest, Hungary; filepne.kovacs.krisztina@uni-mate.hu

**Keywords:** urban ecosystem services, green infrastructure, excessive demand, spatial priority evaluation, urban block

## Abstract

During urbanization in developing countries, fragmentation of green infrastructure due to increasing populations and the expansion of construction land leads to an extremely serious imbalance between the supply and demand for urban ecosystem services. In this study, the central city of Zhengzhou, a central city in central China, was selected as the study area and the excessive demand for six ecosystem services, namely, air purification, flood regulation, heat regulation, hydrological regulation, CO_2_ sequestration and recreational services, was quantitatively evaluated. The entropy method was used to calculate the weights of various ecosystem services, and spatial overlay analysis was performed to obtain the comprehensive ecosystem service excessive demand. Finally, bivariate spatial autocorrelation analysis was used to explore the response of population density to comprehensive excessive demand for ESs. The results of this study indicate that: (1) The most prevalent need is for more CO_2_ regulation service throughout the study area. (2) Except for hydrological regulation service, the spatial distribution of the remaining highly excessive ecosystem service demands are mostly concentrated in old neighborhoods. (3) Of the six excessively demanded economic services, rainwater regulation obtained the greatest weight, reflecting the poor urban infrastructure configuration for countering the rapidly increasing threat of flooding caused by climate change in the city. (4) The comprehensive ecosystem service excessive demand results show that there are eight priority green infrastructure implementation blocks in the central city of Zhengzhou. (5) There were three agglomeration types between population density and comprehensive excessive demand for ESs: high-high type, low-high type and low-low type. The spatial distribution characteristics of population density and comprehensive ES demand are positively correlated. The results of this study could help to provide information for decision making when delineating the priority areas and types of green infrastructure implementation in developing cities.

## 1. Introduction

Ecosystem services (ESs) that directly and indirectly benefit people are partitioned into four different types: provisioning, regulating, cultural and support services [1]. ESs and the natural capital stock that produces them are critical for the functioning and sustainable development of human society. Urban ecosystem services (UESs) can increase urban resilience [2], whereas green infrastructure (GI) is the “natural life support system” that includes all green open spaces around and within the city and constitutes the supply system of UESs [3,4]. UESs depend directly on the quantity, quality and diversity of the GI that produces them, whereas urban ES demand reflects the number of ESs that society expects to receive [2,5].

In the context of rapid urbanization, the continuous expansion of urban construction land has led to a decrease in the size and type of GI and an increase in fragmentation, resulting in a continual decrease in the supply capacity of UESs [4]. Human beings over-consume ecological resources, which causes cities to exhibit a serious imbalance between the supply and demand for ESs in terms of quantity and space. The existing supply and demand configuration of GI does not meet the real ES needs of cities, which is a challenge for current research to determine the capacity of an ecosystem to supply services and the social demand for those services [6]. When the supply of ESs cannot meet the demand, there is an excessive demand for them [7]. Identifying the areas of ecosystem service excessive demand within a city can be used as a priority evaluation method for GI planning to provide a basis for GI construction within a city.

Concerning the research scale, current research related to assessing the supply and demand for ESs is mainly focused on macroregions [7,8,9,10,11,12,13,14]. Although many researchers have made innovative explorations on street-scale GI design [15,16,17,18], the complex dynamic supply and demand mechanism still needs to be combined with urban functional zoning and specific demand groups for more systematic studies. In terms of research methods, the basic idea behind existing ecosystem service supply and demand assessment methods is mainly to calculate and superimpose aggregate supply and aggregate demand separately to identify excessive supply or excessive demand. The quantitative assessment of aggregate supply is usually based on the land use/land cover (LULC) supply or expert scoring calculations [7,19,20]. Quantitative methods and indicators of aggregate demand include unit demand values based on LULC [2], unit demand based on socioeconomic spatial characteristics, the proportion of affected groups or infrastructure [21], and public willingness statistics [22]. When evaluating the spatial allocation of ecosystem service supply and demand, these aforementioned indicators or methods are usually mixed and a single technique is used to identify excessive demand or supply values by directly subtracting the supply and demand values or by normalizing both and then superimposing them. However, the above methods are not applicable to small-scale spatial studies. Since it is usually difficult to balance ecosystem service supply and demand, assessing the spatial allocation by direct superposition and normalization tends to obscure and ignore the absolute ranges of supply and demand, and thus, cannot yield accurate and valuable assessment results.

The main objective of the present study is to explore a quantitative assessment method for direct ecosystem service excessive demand on the urban neighborhood scale in Zhengzhou, and use the assessment results as the basis for the optimization of urban GI construction. At the same time, the research methods can also provide a reference for microscale GI planning of other similar cities. In this study, we take the central area of Zhengzhou City, Henan Province, China as the research area and select the appropriate urban ecosystem service types, as well as the corresponding quantitative indicators and critical thresholds of excessive demand, and conduct a comprehensive quantitative assessment and spatial mapping of its excess demand to obtain the types and spatial distribution of ecosystem service excessive demand in this city. Finally, GIS spatial statistics and bivariate spatial correlation were used to analyze the correspondence between ES excessive demand and population density.

## 2. Materials and Methods

### 2.1. The Study Area

**Figure 1:**
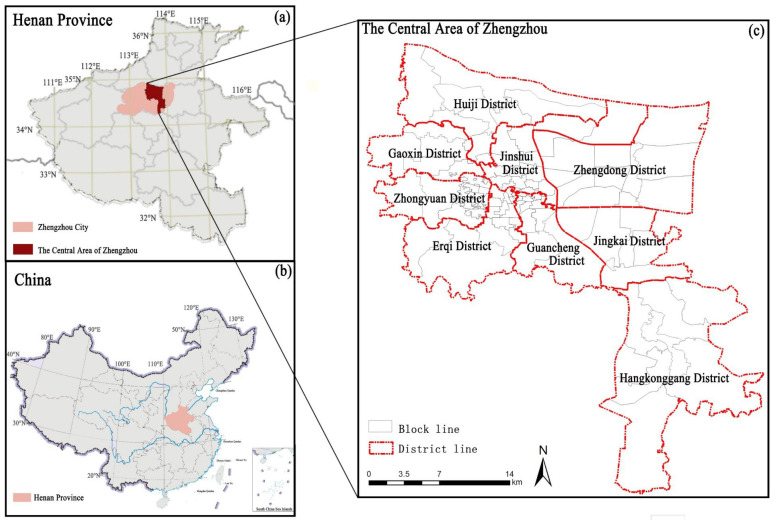
Administrative boundaries (**a**), geographic location (**b**) and topography (**c**) of Zhengzhou city.

Zhengzhou, the capital of the central province of Henan in China, is located at longitude 112°42′–114°14′ E and latitude 34°16′–34°58′ N. The specific area of study is the center of the city (Figure 1). The study area contains 106 streets (townships) with a total area of 628 km^2^, a total population of 5.726 million, and an average population density of 0.9118 million/km^2^.

The climate type of the study area is temperate continental with four distinct seasons, where it has high temperature and rain in the summer and is cold and dry in the winter. The average annual rainfall in the last 10 years was 796.93 mm, with the highest continuous rainfall reaching 624.1 mm in 10 h; the annual maximum temperature is 39 °C, the minimum temperature is −4.2 °C and the maximum urban heat island temperature difference reaches 43.2 °C. In addition, as a developing city since 2000, Zhengzhou has entered a phase of rapid urban expansion and the ecological space within the city has been severely eroded and destroyed. Moreover, natural disasters such as floods, high temperatures and air pollution occur frequently. Due to heavy industrial complexes in the provinces and cities to the north of the city and the influence of the northwest monsoon in the winter, air pollution is significant in the autumn and winter seasons. Based on the ecological and environmental problems occurring in the study area mentioned above, improving the living space within the city, meeting the urban demand for ESs, and increasing the city’s ability to withstand natural disasters are matters that need to be urgently addressed during its current stage of development. Although Zhengzhou has been aware of ecological problems and put forward some improvement measures in recent years, there is no qualitative basis for uneven GI utilization benefits in different regions, especially in the old city, and the problem of too many people and too little green space is very serious. Our study is dedicated to quantitatively assessing the actual demand for ecosystem services on the neighborhood scale and providing a relevant planning and construction basis for the rational allocation of GI.

### 2.2. City Ecosystem Service Types amd Threshold Selection

Urban ecosystem service demand assessment from a GI perspective should take the spatial mobility characteristics of urban ecosystem services, the service supply capacity of GI [3,12] and the demand of the city into account to determine which of the ecosystem services need to be assessed. Our selection of ecosystem service types is based on the following criteria:

(1) GI elements within the city and the demand for urban ecosystem services. For some regulatory services (rainfall, temperature, hydrological regulation, etc.) and cultural services (recreation, spiritual services, aesthetic enjoyment, etc.), the area of GI implementation will determine whether such services can be used effectively.
(2) Concern for stakeholders. For example, persistent haze, the summer heat island effect, and water pollution in Zhengzhou over the past 10 years, especially due to climate change, summer rainstorms and floods, have become predominant problems faced by the city in the past two years that have largely affected its sustainable development.
(3) Policy requirements for carbon emissions. To ensure the implementation of the United Nations 2030 Agenda for Sustainable Development, the country has set the goal of “striving to peak CO_2_ emissions by 2030 and achieving carbon neutrality by 2060”.
(4) Data accessibility. Given the above, we finally selected five ESs (air purification, flood regulation, heat regulation, hydrological regulation and CO_2_ sequestration) and one cultural service (recreational) for excessive demand assessment.

Combined with previous studies [20,23,24,25,26], urban ESs have helped to improve and maintain environmental quality under a certain degree of ecological pressure. However, when the latter exceeds a specified limit, the ecosystem service supply fails to maintain good environmental quality; i.e., ecosystem service demand is not fully satisfied. Therefore, we can use environmental quality standards for the expectation threshold associated with regulating the demand for ESs, and similarly, we can use the corresponding industry normative standards for cultural services, thereby establishing the same critical thresholds for excessive demand.

### 2.3. Data Preparation

Data for the study area included PM2.5 concentrations from the 2020 daily air quality reports from the monitoring stations of the Zhengzhou Ecological Environment Bureau (http://sthjj.zhengzhou.gov.cn, accessed on 9 April 2021), population levels from the 2020 Zhengzhou Statistical Yearbook (https://navi.cnki.net/knavi/yearbooks, accessed on 15 March 2021), days of urban flooding from Landsat 8 OLI remote-sensing data of Zhengzhou in 2020 (http://www.gscloud.cn, accessed on 22 May 2020), surface temperatures from the 2020 Zhengzhou Meteorological Observatory statistics (https://earthexplorer.usgs.gov/, accessed on 11 June 2020), accessibility of regional parks in towns from the park green space of Zhengzhou in 2020 (https://google-earth.gosur.com/cn/, accessed on 20 May 2020), water quality from the report of the Zhengzhou Ecological Environment Bureau on the water quality ranking of the rivers within the city in 2020 (slt.henan.gov.cn/bmzl/szygl/szygb/, accessed on 28 March 2021) and CO_2_ emissions from motor vehicle carbon emission data and 2020 neighborhood green-space carbon absorption data (https://navi.cnki.net/knavi/yearbooks, accessed on 15 May 2021), with excessive data from on-site research (Table 1).

In addition, it should be noted that we used precipitation data from 2021 because Zhengzhou experienced historically rare, heavy rainfall in July 2021, during which the maximum depth of the water reached 3 m and covered more than 95% of the city. The disaster paralyzed the entire city and affected 9 counties and cities downstream. This is a situation that had not occurred in this partially arid inland city in the past century, and thus it is necessary to highlight and add weight to the excessive demand for rainfall regulation in the city in this study.

### 2.4. Quantification Index Selection and Calculation

The frequency and severity of events usually do not fully reflect the magnitude and damage of a disaster, and the exposure and vulnerability of the city to a disaster need to be considered simultaneously. Therefore, to quantify the risk of disaster (risk, R), we selected the disaster risk assessment framework proposed by the Intergovernmental Panel on Climate Change (IPCC) based on “Hazard-Exposure-Vulnerability” [27], as expressed in the following equation:

(1) R=H×E×V

where *R* is the corresponding disaster risk when hazard *H* exceeds the critical threshold, exposure *E* is the direct exposure carrier of the disaster and vulnerability *V* is the ability of the affected object to withstand or cope with the disaster. We calculated the selected indicators based on the disaster assessment framework in the evaluation of the following six ESs.

#### 2.4.1. Air Purification

Due to the influence of the geographical location and seasonal climate, air pollution in the study area is the highest in winter and alternates in the winter, spring and autumn–winter seasons. Haze is the predominant problem affecting urban air quality in the area, with the primary pollutant being PM_2.5_. Therefore, we used the PM_2.5_ pollution risk index as an excessive demand evaluation indicator for air purification. The risk index should be used to measure vulnerability in socioeconomic terms more comprehensively than by simply using indicators such as concentration or the number of occasions that the pollution limit is exceeded, because the lower the vulnerability the stronger the demand for the ecosystem service. Therefore, we used PM_2.5_ concentration, population density and social vulnerability as secondary indicators (Table 1). Firstly, we used the annual average PM_2.5_ concentration limit (35 μg/m^3^) specified in the Ambient Air Quality Standards (GB 3095-2012) as the excessive demand threshold. The selection of the annual average indicator for evaluation can reflect the ecosystem service demand levels for different neighborhoods, and the selection of extreme values can affect the actual objectivity of the assessment results. By using a reclassification tool, areas with annual average PM_2.5_ concentrations of less than 35 μg/m^3^ were classified as 0, whereas other areas were classified into five levels from 1 to 5 using the natural breakpoint method (with “1” indicating the lowest level of stress and “5” indicating the highest level of stress) as the stress index. The population density and the proportions of the elderly and children in the population on the street scale within an urban area were similarly reclassified and assigned values from 1 to 5, which were used as the exposure evaluation index and the social vulnerability evaluation index, respectively.

Subsequently, we normalized the indicators as follows:

(2) $$x^{\prime }=\frac{x-x_{\min}}{x_{\max}-x_{\min}}$$

where *x* is the original value, $x^{\prime }$ is the normalized value, $x_{\min}$ is the minimum value and $x_{\max}$ is the maximum value.

Finally, according to the risk assessment framework in Equation (1), the PM_2.5_ risk index for the study area was obtained and reclassified using six levels from 0 (no excessive demand) to 5 (the most excessive demand).

#### 2.4.2. Flood Regulation

As a relatively arid inland city, Zhengzhou has rarely experienced heavy rainfall or flooding in the past 50 years. However, since 2000, the rapid expansion of the city size and the inability of infrastructure development to meet the demands of urban development has resulted in frequent and multi-scale flooding within the city during rainfall events. Coupled with global climate change and environmental deterioration, the city has been highlighted by heavy rainfall and flooding in the past two years. Therefore, we selected the flood risk index as a quantitative indicator to assess the excessive demand for a rainfall and flood regulation service in urban neighborhoods within the study area and selected the simulated waterlogging depth, the quantity of affected infrastructure and population, and social vulnerability as secondary indicators (Table 1). Satellite remote-sensing monitoring technology was used to obtain the inundation extent, and the ArcGIS spatial analysis and data management functions were applied to obtain the inundation depth in combination with the ground elevation data of the study area to obtain the flood risk index. According to the evaluation criteria of the inundation level in the Planning Standards for Urban Inundation Prevention and Control issued by the Ministry of Housing and Urban-Rural Development, a water depth of 15 cm on a road was taken as the excessive demand threshold for the rainfall regulation service. The disaster-bearing bodies for an internal flooding disaster, including road network density, building area density and population density, were selected for the exposure index evaluation.

#### 2.4.3. Heat Regulation

The prominent environmental problem in the study area in summer is urban high temperature with a significant heat island phenomenon. The maximum daily temperature in urban areas reached 39 °C in 2020, and the temperature in many places is 6–8 °C higher than in suburban areas. We selected surface temperature, population density and social vulnerability as secondary indicators to construct an urban high-temperature risk assessment index system to assess excessive demand for heat regulation services (Table 1). According to the group standard for climate recreational site evaluation issued by the National Weather Service Association and the three levels of high-temperature warning signals established by the meteorological department for hot weather (yellow, orange and red warnings mean that the maximum temperature will be above 35 °C for 3 consecutive days, rise to 37 °C within 24 h and rise to 40 °C within 24 h, respectively), we selected 35 °C as the threshold value for the temperature regulation service demand in this evaluation.

We selected the Landsat 8 thermal infrared remote-sensing band for a day in summer 2020 and used the ENVI remote-sensing processing software to perform remote-sensing inversion of the surface temperature in the study area by employing the atmospheric correction method. The surface temperature obtained from the inversion was then reclassified to obtain the corresponding high-temperature risk disaster-stress index. The exposure and vulnerability indices were calculated in the same way as for the air purification service.

#### 2.4.4. Hydrological Regulation

We selected the water quality security index as a quantitative index to assess the excessive demand for the urban water hydrological regulation service. We graded the water bodies in the urban districts in the study area that did not reach the excellent standard from 1–5 according to the report of the Zhengzhou Ecological Environment Bureau on the water quality ranking of the rivers within Zhengzhou in 2019, whereas neighborhoods without water bodies were graded as 0. The index was calculated by using the China surface water environmental quality class 3 standard (GB3838-2002) and urban sewage treatment plant pollutant discharge class 1 A standard (GB18918-2002). For local water bodies where the water quality was not being monitored (e.g., rivers and local water runoff areas with intermittent flow during particular months), we conducted on-site research and supplemented the missing data.

#### 2.4.5. CO_2_ Sequestration

We selected the CO_2_ emission risk index as the quantitative indicator of excessive demand for the CO_2_ regulation service, and CO_2_ emission, population density and social vulnerability as secondary indicators (Table 1), indicating the degree of stress hazard , exposure index and the proportion of the elderly and children in the population, respectively. Because the scope of this study involves the inner city on the neighborhood scale, we added together the carbon emissions from people and motor vehicles and carbon sequestration from green spaces as the total carbon emissions (Equations (3)–(6)). By combining the data from the IPCC Technical Report and Methodological Guidelines [28] and previous studies [29,30], the carbon emissions per vehicle per year for motor vehicles were assumed to be 2.7 t, the CO_2_ exhalation per person per year was assumed to be 0.079 t, and the carbon emission factor for urban green spaces was assumed to be −5.77 t/(hm^2^·a).

Carbon emissions from motor vehicles can be calculated by using:

(3) $$C_{e}=\sum (P_{n}\times \theta_{n})$$

where $C_{e}$ denotes the CO_2_ emissions from $P_{n}$ motor vehicles in the region and $\theta_{n}$ denotes the CO_2_ emissions per motor vehicle per year.

Carbon emissions from human respiration can be calculated using the following formula:

(4) $$C_{p}=\sum (P_{i}\times \theta_{i})$$

where $C_{p}$ denotes the CO_2_ emissions per person in the region,$\;P_{i}$ denotes the number of people in the region and $\theta_{i}$ denotes the CO_2_ emissions per person per year.

The equation for calculating carbon emission in urban green spaces is as follows:

(5) $$C_{l}=\sum (S_{i}\times \delta_{i})$$

where $C_{l}$ indicates the CO_2_ uptake in the green space, $S_{i}$ indicates its area and $\delta_{i}$ indicates the rate of carbon sequestration per unit area of the green space.

Thus, total carbon emissions can be calculated as:

(6) $$C=C_{e}+C_{p}+C_{l}$$

Due to limited available data, the population and motor vehicle carbon emissions used are not representative of all carbon emissions in the study area; thus, we used the natural breakpoint method to directly classify the processed data into levels from 0–5.

#### 2.4.6. Recreational Services

Accessibility is an important indicator affecting the degree of urban green-space use that reflects the ease with which residents can overcome spatial resistance to approach and use park land and the relationship between urban green space and potential use demand within a regional unit [31,32]. We used open space opportunities as a primary quantitative indicator of excessive demand and the number of people with low open space opportunities as a secondary indicator. We applied use of the point-of-interest (POI) method to obtain the base data for urban neighborhood discrimination and location data for green spaces by cleaning and classifying the POI data and using ArcGIS to obtain the park service capacity for the total green-space area in the neighborhood. To take the “boundary effect into consideration”, we included neighboring parks outside the study area in the analysis process. According to the “Service Radius Classification Requirements for Urban Parks”, an excessive demand threshold of 15 min was used as the criterion for good accessibility. The number of people in the low accessibility range in each neighborhood was obtained by superimposing the population density. Finally, the excessive demand for recreational services was reclassified from 0–5.

### 2.5. Weighting Calculation

We determined the weights for each excessive demand from the perspective of overall fairness based on measuring the discrete degree of each excessive demand index using MATLAB software and the entropy method in the objective assignment method. The specific calculation steps are as follows.

Normalize the indicators:

(7) $$x_{ij}=\frac{D_{ij}-D_{j\min}}{D_{j\max}-D_{j\min}}$$

Calculate the share of excessive demand for the ecosystem service item *j* in block *i*:

(8) $$X_{ij}=\frac{x_{ij}}{\sum_{i=1}^{x}x_{ij}}$$

Calculate the information entropy of each requirement:

(9) $$e_{j}=-\frac{1}{\ln m}\overset{m}{\underset{i=1}{\sum }}X_{ij}\ln X_{ij},e_{j}\in \left[0,1\right]$$

Calculate the information entropy redundancy:

(10) $$d_{j}=1-e_{j}$$

Calculate the weight of each excessive demand:

(11) $$W_{j}=\frac{d_{j}}{\sum_{i=1}^{n}d_{j}}$$

Based on the weights, the comprehensive ecosystem service excessive demand value for each neighborhood can be calculated as:

(12) $$S_{i}=\overset{n}{\underset{j=1}{\sum }}W_{j}x_{ij}$$

In Equations (7)–(12),$\;D_{ij}$ is the value of the *j*th ecosystem service demand in the *i*th block; $D_{j\max}$ and $D_{j\min}$ are the maximum and minimum values of the matrix column in which the *j*th ecosystem service excessive demand is located, respectively; *m* is the number of blocks and *n* is the total number of ecosystem service excessive demands evaluated.

### 2.6. Bivariate Moran’s I Calculation

After preliminary analysis, we found that the spatial numerical distribution of comprehensive ES excessive demand and population had a certain degree of spatial autocorrelation. Bivariate spatial autocorrelation has high applicability and effectiveness in describing the spatial correlation and dependence characteristics of two geographic elements [33]. At present, this method has not been found to be used to explore the spatial relationship between ES demands and population distribution. Therefore, we innovatively attempted to adopt this method.

Bivariate Moran’s I is an extension and expansion based on Moran’s I index, which measures the correlation between the attribute values of spatial units and other attribute values in adjacent spaces [34,35]. It can be used as an effective method to analyze the correlation characteristics between comprehensive UES demand and population density. Bivariate Moran’s I is divided into two levels: global Moran’s I and local Moran’s I. The calculation formula is as follows:

(13) $$I_{ab}=\left(\frac{x_{ma}-{\bar{x}}_{a}}{\delta_{a}}\right)\left(\frac{x_{ab}-{\bar{x}}_{b}}{\delta_{b}}\right)\overset{n}{\underset{j=1}{\sum }}w_{mo}$$

In Equation (13), $x_{ma}$ is the value of the variable *a* of the spatial unit *m*, $x_{ab}$ is the value of the variable *b* of the spatial unit *o*, ${\bar{x}}_{a}$ and ${\bar{x}}_{b}$ are the mean values of *a* and *b*, respectively, $\delta_{a}$ and $\delta_{b}$ are the variances of *a* and *b*, $w_{mo}$ is the spatial weight matrix between unit *m* and *o*, and $I_{ab}$ is the Moran’s I statistic and its value is between (−1, 1): less than 0 means negative correlation, equal to 0 means no correlation and greater than 0 means positive correlation. Data processing was conducted in GeoDa 1.6.7.

## 3. Results

### 3.1. Excessive Demand Evaluation for Each UES

**Figure 2:**
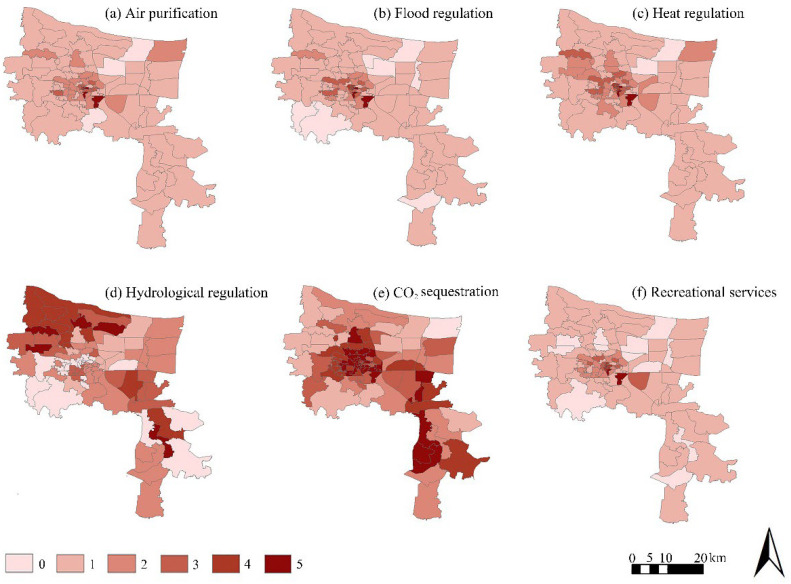
The results of excessive demand evaluation for each urban ecosystem service.

Figure 2 shows the evaluation results for each ecosystem service excessive demand. The numbers of blocks with the air purification service excessive demand from 5 to 0 were 3, 2, 7, 21, 58 and 1; there are obvious differences in the spatial distribution, with the areas having high excessive demand being concentrated in the old blocks with high populations and building densities in Guancheng District, including Beixia, Nanguan and East Hanghai blocks. Guancheng District is the old city of Zhengzhou, with dense buildings and population, and a lack of green space, including the Beixia block with an area of 1.2 km^2^, a population of 89,886, and a green area of 0.017 km^2^; the Nanguan block with an area of 1.7 km^2^, a population of 43,884, and a green area of 0.078 km^2^; and the East Hanghai block with an area of 8.6 km^2^, a population of 39,043, and a green area of 0.7 km^2^. The above three blocks are mostly covered by small roads, and there is almost no three-dimensional greening and rooftop greening, thus it is difficult to improve the air quality effectively.

The numbers of blocks with excessive demand for flood regulation from 5 to 0 were 3, 4, 9, 12, 59 and 1; the numbers of blocks with excessive demand for heat regulation from 5 to 0 were 3, 5, 12, 52 and 1. The results for the high-demand neighborhoods are the same as for air purification, indicating that not only are the dense building population and the extreme lack of green-space resources a problem, but also the various infrastructures for drainage and the construction of pavements and buildings in such old neighborhoods are relatively outdated and unable to meet the requirements of energy efficiency and environmental protection, so much so that they cannot meet the needs of current urban development. 

The numbers of blocks with excessive demand for hydrological regulation from 5 to 0 were 5, 9, 16, 31, 10 and 1. Wutong and Shuangqiao blocks in Gaoxin District, Yingbin and Yangjin blocks in Huiji District, and the Binhe block in Hangkonggang District have high demand. Among these areas, Wutong, Shuangqiao and Yingbin blocks have black smelly water bodies, and the rest of the blocks are located in the middle and lower reaches of urban rivers where some of their river water quality classes are monitored as V. That is the main reason for the high overall demand value of hydrological regulation of the blocks. 

The numbers of blocks with excessive demand for CO_2_ sequestration from 5 to 0 were 30, 21,14, 13, 15 and 1. The results in Figure 2 show that CO_2_ regulation is the indicator with the highest number of high-demand neighborhoods among all single ES demand services, and the demand for CO_2_ regulation has the widest spatial distribution, which reflects the serious inequality between carbon emissions and carbon sequestration within the city. The high-demand neighborhoods are mainly located in Jinshui District (Fengqing, Nanyangxincun, Nanyang, Dashiqiao, Wenhua, Jingba, Fengchan and Fenghuangtai blocks), Zhongyuan District (Mianfang, Jianshe and Linshanzhai blocks), Eerqi District (Wulibao, Minggong, Mifengzhang, Jiefang, Dehua, Yima, Jianzhong and Huaihe blocks), Guancheng District (Xidajie, Nanguan, Dongdajie, Longhai, Erligang and East Hanghai blocks), Jingkai District (Jinghang block), which is located in the southern part of the new city near the surrounding industrial land, and Hangkonggang District (Zhenggang, Xingang and Yinhe blocks). The high demand for CO_2_ regulation in the old city is directly related to the high density of human life and motor vehicle emissions, as well as the small and sporadic distribution of the GI area, which makes it difficult to neutralize the continuously increasing CO_2_ emissions. The main reason for the high demand for CO_2_ regulation is the proximity of these neighborhoods to industrial areas and expanding construction areas on the outskirts of the city, (although we did not count the CO_2_ emissions from the industry here) the high motor vehicle emissions from industrial and construction transportation, and the lack of GI land for CO_2_ absorption in these neighborhoods. The GI land area of the Jinghang block is 0.0001 km^2^, and the GI land areas of Zhenggang, Xingang and Yinhe blocks are 7.06 km^2^, 9.59 km^2^ and 14.52 km^2^, respectively, thus the final calculation shows that the excessive demand is high. 

The numbers of blocks with excessive demand for recreational services from 5 to 0 were 3, 5, 8, 15, 51 and 1. As can be seen from Figure 2, the excessive demand for recreation services is more concentrated in the south of Jinshui District, the east of Zhongyuan District, and the north of Erqi and Guancheng Districts, which are among the earliest developed central urban areas of Zhengzhou City where the green infrastructure and recreation space allocated at the beginning of development were not serious considerations of the government at the time of planning and construction; therefore, there is woefully inadequate public open green space. In particular, the Dehua block in Erqi District and Nanguan and East Hanghai blocks in Guancheng District have the highest demand for recreation services in terms of walking arrival time.

### 3.2. Evaluation of the Comprehensive Excessive Demand for UESs

**Figure 3:**
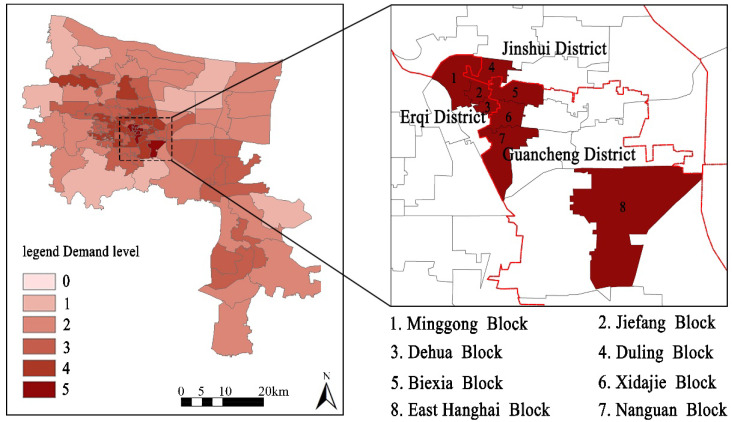
Evaluation of the comprehensive excessive demand for UESs.

According to the weighted calculation results, excessive demand for flood regulation had the largest weight of 0.406, followed by air purification (0.305), heat regulation (0.119), recreational services (0.074), CO_2_ regulation (0.073) and hydrological regulation (0.021). ArcGIS software was used to comprehensively stack the obtained weight values and obtain the comprehensive ecosystem service excessive demand after recalculating the classification (Figure 3). The results show that the numbers of blocks with comprehensive ecosystem service excessive demand values from 5 to 0 were 8, 21, 31, 25, 9 and 0, among which there were eight with particularly high excessive demands: Minggong, Jiefang and Dehua blocks in Erqi District; Duling block in Jinshui District; and Beixia, Xidajie, Nanguan and East Hanghai blocks in Guancheng District. (Table 2). From the spatial structure perspective, the areas with high excessive demands are mainly distributed in the old blocks in the city, whereas the blocks with low excessive demands are mainly distributed in the areas near the urban edge or new urban areas.

Improving the GI unit efficiency supply regulation should be based on comprehensive demand rather than on a single benefit or purpose. The above eight blocks with the highest excessive demands for comprehensive ESs should be prioritized for GI construction in the central city of Zhengzhou in the future. It should be emphasized that using the comprehensive ES excessive demand identifies high-priority areas for GI implementation, whereas the specific implementation type of GI is obtained by analyzing each excessively demanded ES. First of all, according to the comprehensive ES excessive demand results obtained by weighted superposition, the eight blocks with high comprehensive ES excessive demand can be set as priority implementation areas for GI, after which the corresponding single excessively demanded ES for each area can be analyzed. For instance, the ecosystem service excessive demands for the Xidaijie block follow this pattern: CO_2_ regulation (5) > recreational services (4) and heat regulation (4) > air purification (3) = flood regulation (3) > hydrological regulation. Therefore, CO_2_ regulation should be the primary goal for the future GI planning of this street. Overall, among the eight blocks with high, comprehensive ecosystem service excessive demand, CO_2_ regulation ranks first, which also indicates that for the GI construction for the central city of Zhengzhou, priority should be given to reducing CO_2_ emissions in the future. In addition, the improvement of urban air quality, prevention of flooding and dealing with urban high temperatures are second only to the reduction of CO_2_ emissions for the implementation of GI.

### 3.3. Bivariate Spatial Autocorrelation Analysis

**Figure 4:**
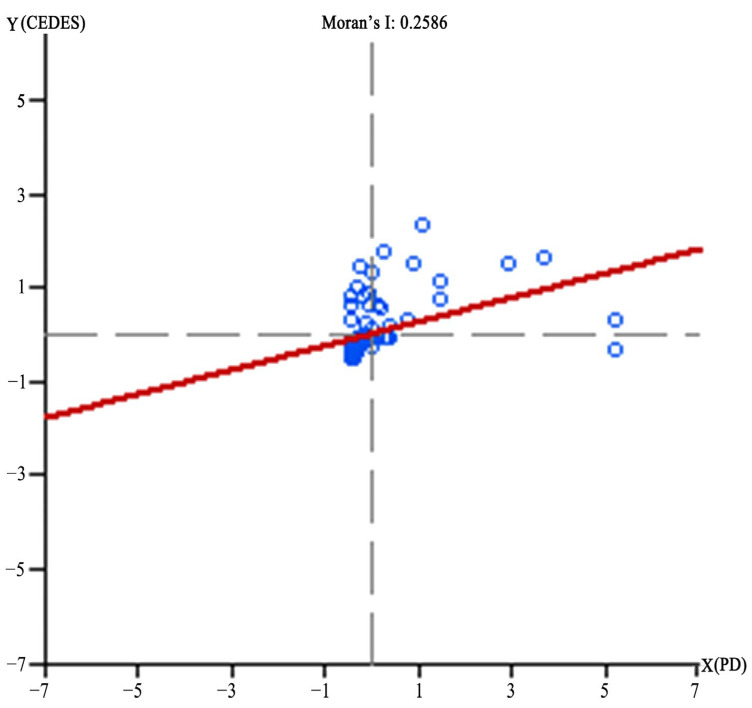
The Moran’s I scatter diagram of population density and comprehensive excessive demands for ESs.

Using GeoDa software, the bivariate spatial autocorrelation analysis was carried out with population density (PD) as the first variable (X) and comprehensive excessive demands for ESs (CEDES) as the second variable (Y). The global Moran’s I was 0.259 (Figure 4). Randomization 999 was selected in GeoDa for the significance test. The results showed that the p values were all 0.001, indicating a significant spatial positive correlation between LST and habitat quality under the confidence of 99.9%; that is, with the increase in population density, the comprehensive excessive demand for ESs in the center of Zhengzhou also increases.

**Figure 5:**
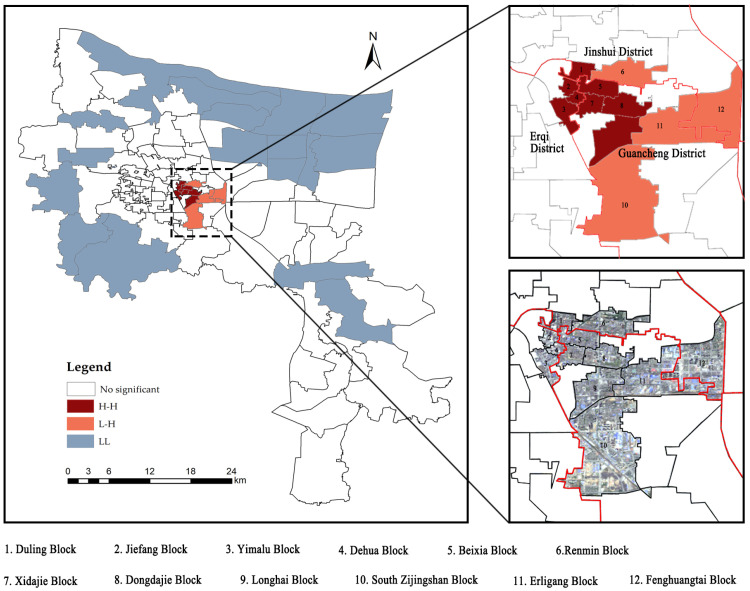
Local indicators of spatial association cluster map of population density and comprehensive excessive demand for ESs.

The global Moran’s I only represents the overall correlation trend of the two variables and cannot reflect the agglomeration differences in specific spatial locations. Therefore, the local Moran’s I was further calculated, and the results (LST-habitat quality) were divided into three aggregation types: high-high (H-H), low-high (L-H) and low-low (L-L). The LISA (local indicators of spatial association) cluster map of population density and comprehensive excessive demand for ESs clearly shows the spatial agglomeration characteristics of regions that passed the significance test (Figure 5).

The H-H type is scattered in Erqi District and Guancheng District. Erqi District includes Duling, Jiefang, Yimalu and Dehua blocks; Guancheng District includes Beixia, Xidajie, Dongdajie and Longhai blocks. The above areas are located in the center of the old blocks in Zhengzhou, with typical characteristics of high population density, high building density and very little GI distribution. Among these blocks, the highest population density is found in the Dehua block, which is 162,325 persons/km^2^, and the lowest is in the Longhai Road block, with 18,711 people/km^2^. Their high demand for ESs is reflected in all aspects of GI configuration, including improving air quality, reducing waterlogging, lowering summer temperatures, increasing recreational space, etc.

The L-H type is distributed in Guancheng District and Jinshui District. Guancheng District includes South Zijingshan and Erligang blocks; Jinshui District includes Renmin and Fenghuangtai blocks. The population density is 9274 people/km^2^ in the South Zijingshan block, 7244 people/km^2^ in the Erligang block, 16,381 people/km^2^ in the Renmin block and 11,137 people/km^2^ in the Fenghuangtai block. The reasons for the relatively low population density and high additional demand are the high traffic flow in the South Zijingshan block and the excessive building density in the residential area; the Erligang block, Renmin block and Fenghuangtai block’s inclusion is mainly caused by the high demand for CO_2_ regulation.

The L-L type is mainly distributed in the fringes of the central urban area. These blocks were developed late, with low population density and relatively complete infrastructure. The layout of buildings and roads in the blocks is more reasonable. In addition to the surrounding suburbs, there are large areas of green space to reduce the risk of disasters in these areas.

## 4. Discussion

### 4.1. Excessive Demand Evaluation for Each UES

At present, the versatility of GI has been widely accepted and recognized, but research into and implementing it are often carried out to bestow a single benefit [36]. This ignores the overall benefits of GI to some extent and is not conducive to the efficient use of social capital. In addition, most of the benefits generated by GI are highly localized and located in or near supply areas, making decisions on their allocation important for local environmental and social equity. This is why we assessed several ES needs, including regulation services and cultural services, separately.

Taking the central area of Zhengzhou as an example, we conducted a single quantitative evaluation and spatial mapping on six excessively demanded ESs (air purification, flood regulation, heat regulation, hydrological regulation, CO_2_ regulation and recreational services) based on environmental quality standards with the block as the basic unit. The results show that:

(1) Comparing the evaluation results of the six indicators, except for hydrological regulation and CO_2_ regulation, the other four indicators show that the high-demand areas are located in the old urban areas, which were the first to be developed but now have poor environmental conditions. Therefore, in terms of supply, old urban areas need to be equipped with GI facilities that can solve air pollution, waterlogging, high temperature and lack of open space, and the corresponding solution strategy needs to be based on the construction characteristics of each neighborhood, such as adding three-dimensional greening and green roofs for buildings that meet the implementation goals. For example, the rational design of road space and pavement, with rainwater gutters and permeable paving materials that can both increase the road space and pavement can be reasonably designed, and the rain gutters and permeable paving materials can not only increase the water permeability and water storage but also increase the planting space; furthermore, small, abandoned spaces around the streets can be used to create recreational places and so on.
(2) The evaluation of hydrological regulation in this study focuses on the water quality of rivers and public-space landscape lakes in urban areas; there are neighborhoods without river or lake distribution and these areas cannot be evaluated, thus their demand value is 0. As a result, in the calculation process of evaluating the comprehensive ES demand, this indicator has the lowest weight value, which has no influence on the final results. From the final evaluation results, the water environment quality in the areas located in the northwest and southeast of the central city is poor, mainly because: firstly, there are more rivers in the northwest of the city, but the construction along the rivers is lagging behind, there is a lack of coherent green space, and there is more unused land distribution, which makes it easy to have garbage accumulation or sewage discharge; secondly, the southeast of the city is located downstream of the city rivers, plus the area is in the expansion and construction stage and there is a certain industrial distribution in the periphery, which makes the water body more vulnerable to pollution. The implementation of GI in the river area should focus on increasing the spatial coherence and the purification effect of plants on water bodies, as to form an urban greenway combining blue and green spaces.
(3) Combined with the evaluation results of CO_2_ regulation, the central old city and the new city in the southeast, as the high-value areas of CO_2_ emissions, are influenced by the daily activities of residents and construction and industrial development, respectively. Based on the above factors, firstly, in the neighborhoods with high CO_2_ emissions in the old city, the implementation of GI should focus on increasing the planting space that can collect CO_2_ as much as possible while focusing on the use of relevant energy-saving facilities; secondly, for the economic development zone and aviation port area in the southeast, there is a larger area of space for GI construction, and thus it is necessary to reserve enough land for GI and build energy-saving and emission-reducing green buildings at the early stage of planning and construction.

### 4.2. Comprehensive Excessive Demand for UESs

Based on the results of the individual ES demand evaluation, we then assessed the additional demand for comprehensive ESs in each neighborhood within the central city of Zhengzhou. The purpose of the comprehensive ES demand evaluation was to identify priority areas for GI implementation, which helps decision-making authorities to make decisions with limited financial and resource support that are more equitable to the local environment and society. Compared with previous studies on ecosystem services [37,38], we paid more attention to the practicality of comprehensive demand of ES supply and demand in urban GI planning and construction. For example, Morse Wayde C et al. explored the relationship between outdoor recreation and ecosystem services, and Ruchira Gangahagedara et al. proposed research trends and research priors for ESs related to multidirectional biodiversity and climate change. Based on the results of this study, the following optimization measures about the implementation of GI are proposed for the future:

(1) In terms of spatial priority, the results of excessive ES demand after comprehensive superposition indicate that there are eight high-demand blocks in total, and all of them are clustered at the junction of Guancheng District and Erqi District. These blocks belong to the earliest developed areas in Zhengzhou. Therefore, the implementation of GI should give priority to old neighborhoods and old city renewal. Here, we propose two preliminary strategies: hard GI and soft GI. First, hard GI includes the use of permeable and water-storage materials in the road pavement and energy-saving facilities in buildings, etc. Hard GI is an effective supplement to the original, backward infrastructure in the neighborhood that enhances the drainage function inside the streets and reduces CO_2_ emissions and excessive use of energy. Secondly, soft GI includes green space mainly for planting. On the one hand, in the old neighborhoods with extremely limited land space there are small spaces with poor or unreasonable utilization, such as street corners and street edges, in which GI layout can be utilized in the form of pocket parks and where miniature dotted and strip planting can be used for renewal and renovation; on the other hand, three-dimensional greening and rooftop greening can be added to buildings and structures with feasibility. In short, the scattered layout of soft GI in the form of “stitching” can improve the neighborhood air quality, reduce the heat island effect of small-scale space, absorb waterlogging and meet the residents’ recreational needs with maximum efficiency. In addition, enhancing and maintaining the development and the quality of the green infrastructure in the blocks should not be ignored, and improving the development level of green infrastructures such as rivers, parks and road green belts can enhance the function of ESs such as hydrology and climate in the blocks.
(2) In terms of the excessively demanded ESs for the center of Zhengzhou, the decision-makers should first consider the supply of GI to improve the CO_2_ regulation service. There is a close relationship between carbon emissions and air quality. The reason for this result is directly related to the backward energy-saving facilities, excessive population and building density in old blocks. Therefore, in addition to the above measures, a more in-depth study of tree species selection and enhanced connectivity for non-motorized mobility within the GI could increase CO_2_ uptake by plants and improve the convenience of low-carbon travel for the population to some extent.

### 4.3. Bivariate Spatial Autocorrelation Analysis

We conducted a spatial autocorrelation analysis of population density and comprehensive ES demand. The calculation results of the Moran’s I and LISA cluster map can help us to analyze the reasons for additional ES demand in different neighborhoods in the city more thoroughly and can scientifically verify the demand types of GI calculated by the established ES excessive demand evaluation system and the areas in urgent need of priority construction. For example, it can be seen from the analysis results that although population density and ES demand have the most direct correlation, high-demand and high-population density blocks do not equate. Therefore, we should focus on four areas of GI functionality configuration:

(1) For H-H type blocks, GI should be allocated based on low per capita resources and small available space, and environmental issues directly related to people’s health should be primarily solved, such as improving air quality and alleviating high temperatures in summer.
(2) For L-H type blocks, due to the relatively low population density, GI configuration has a large role to play. Therefore, a GI supply strategy should be proposed for these blocks one by one, based on the single ES demand assessment results of each block.
(3) For L-L type blocks, which are mainly distributed in new areas around central urban areas, the population density and demand for ecosystem services are relatively low. However, our on-the-spot investigation found that a large amount of green infrastructure, such as street green spaces, greenways and comprehensive parks, in these blocks is inefficiently utilized. Therefore, GI configuration needs to pay attention to individual blocks with high demand for a single ES and be based on functional supplement.
(4) For blocks with high ES demand not reflected in the above three types, such as Minggong, Nanguan and East Hanghai blocks, we need to further analyze the main reasons for high ES demand and propose corresponding GI supply strategies according to the results of individual ES evaluation.

## 5. Conclusions

The aim of the present study was to provide effective auxiliary information to aid decision-making on GI from two aspects: the implementation area and the type of GI. Based on the above research, the conclusions of this paper are as follows:

(1) We attempted to establish a new evaluation system in the context of the mismatch of urban GI supply and demand and use environmental quality standards and industry code standards to establish excessive demand thresholds for ESs. We demonstrated the applicability of the evaluation system in regulation services and recreation services.
(2) Our evaluation method can effectively maximize the urban GI configuration by providing intuitive quantitative results to reflect the actual demand for ESs in urban areas and improve the ability of urban areas to resist natural disasters. The assessment method based on establishing excessive demands for ESs could be applied to other ecologically fragile ecological areas affected by urban development and climate change.
(3) In the implementation of GI, many researchers provide measures to enhance resilience and prevent natural disasters. For example, Daeyoung Jeong et al. proposed a GI planning strategy for disaster prevention and evacuation in coastal cities [39]. We recommend that more quantitative environmental assessments be incorporated into planning, particularly in GI planning.
(4) The present study has some limitations. Firstly, we did not consider subjective service needs outside of the ecological environment, such as aesthetics, historical and cultural heritage, etc. Secondly, our assessment method is for excessive demand for ESs on the urban scale. However, in practice, green-space planning on only one scale may not enable full realization of the effect of providing GI on a larger scale. In addition, In this study, we only analyzed the autocorrelation between population density and comprehensive ES demand. Due to the limitations of collectible data, etc., there was no one-by-one analysis between population density and ES individual indicators. In the future, we will continue to study the internal driving mechanism quantitatively and combine corresponding engineering techniques to help maximize the ecological benefits of GI. For example, in old, built-up urban areas, GI supplements should be carried out by planting landscapes such as roof gardens, vertical greening and street green spaces, and GI such as permeable roads and drainage facilities should be improved or increased in areas where conditions permit. In later expansion areas, the GI construction should mainly focus on increasing recreational places and green land, improving water-permeable facilities in large areas, and encouraging the large-scale implementation of roof greening and vertical greening in new buildings.
(5) In the follow-up study, we will improve the evaluation system from two aspects: on the one hand, we will further consider adding more evaluation indicators, such as biodiversity, travel efficiency, urban style, etc. On the other hand, we will further explore the internal mechanism of high and low imbalance, including how to regulate population from the planning scale, the developmental direction of built-up areas, the construction of the interconnected ecological network, etc. Therefore, our next task is to consider how to carry out large-scale and multi-level ecosystem service assessments and GI planning in the context of territorial space planning, with the focus on smaller-scale convergence and coordination, which is also an important challenge in this field of research.

The above research results can help the city stakeholders to prioritize green spaces and categories of GI implementation and provide more objective auxiliary information for decision-making in regard to the planning and implementation of GI. Our method augments GI allocation for green-space system planning by taking background socioeconomic factors into account and helps to maximize the comprehensive ecosystem service benefits provided by GI.

## Figures and Tables

**Table 1:** ES excessive demand evaluation indicators and demand thresholds.

ES	Indicators	Secondary Indicators	Excess Demand Threshold	Data
Air purification	PM_2.5_ pollution risk index	PM_2.5_ concentration	35 μg/m^3^	Daily average PM_2.5_ concentration data at monitoring stations in Zhengzhou in 2020
		Population density	-	Population statistics by neighborhood in Zhengzhou in 2020
		Social vulnerability	-	Share of elderly and child population
Flood regulation	Flood risk index	Simulated water damage depth	15 cm	Modeled waterlogging depth data for 2020; rainfall statistics for 2021
		Number of affected infrastructure and population	-	Remote-sensing data on urban buildings and roads; 2019 population statistics by neighborhood in Zhengzhou
		Social vulnerability	-	Share of elderly and child population
Heat regulation	High-temperature risk index	Surface temperature	35 °C	Remote-sensing image data for Zhengzhou City in 2020
		Population density	-	Population statistics by neighborhood in Zhengzhou in 2020
		Social vulnerability	-	Share of elderly and child population
Hydrological regulation	Water quality safety index	Average annual water quality	Excellent	Zhengzhou Ecological Environment Bureau Report on Water Quality of Rivers in Zhengzhou City in 2019
		Population density	-	Population statistics by neighborhood in Zhengzhou in 2020
		Social vulnerability	-	Share of elderly and child population
CO_2_ sequestration	CO_2_ emission risk index	CO_2_ emission	-	Neighborhood population carbon emissions data in 2020; neighborhood green-space carbon sequestration data in 2020
		Population density	-	Population statistics by neighborhood in Zhengzhou in 2020
		Social vulnerability	-	Share of elderly and child population
Recreational services	Low recreational opportunity population	Park accessibility	15 min	Park land and road vector data for Zhengzhou in 2020
		Population density	-	Population statistics by neighborhood in Zhengzhou in 2020

**Table 2:** High, comprehensive ES excessive demand blocks.

ES High-Value Neighborhood	District	Air Purification	Rainfall Regulation	Temperature Regulation	Hydrological Regulation	CO_2_ Regulation	Recreational Services
Jiefang	Erqi District	4	4	4	1	5	4
Nanguan	Guancheng District	5	5	5	2	5	5
East Hanghai	Guancheng District	5	5	5	2	5	5
Beixia	Guancheng District	5	5	5	2	4	4
Dehua	Erqi District	4	4	4	2	5	5
Xidajie	Guancheng District	3	3	4	2	5	4
Minggong	Erqi District	4	4	4	0	5	3
Duling	Guancheng District	4	4	4	0	5	4

## Data Availability

The data presented in this study are available on request from the first author.

## References

[1] Corvalan C., Hales S., McMichael A., Butler C., Campbell-Lendrum D., Confalonieri U., Leitner K., Lewis N., Patz J., Polson K. (2005). Ecosystems and Human Well-Being: Health Synthesis.

[2] Calderón-Contreras R., Quiroz-Rosas L.E. (2017). Analysing Scale, Quality and Diversity of Green Infrastructure and the Provision of Urban Ecosystem Services: A Case from Mexico City. Ecosyst. Serv..

[3] Benedict M.A., McMahon E.T. (2002). Green Infrastructure: Smart Conservation for the 21st Century. Renew. Resour. J..

[4] Wang Y.-C., Shen J.-K., Xiang W.-N. (2018). Ecosystem Service of Green Infrastructure for Adaptation to Urban Growth: Function and Configuration. Ecosyst. Health Sustain..

[5] Schröter M., Barton D.N., Remme R.P., Hein L. (2014). Accounting for Capacity and Flow of Ecosystem Services: A Conceptual Model and a Case Study for Telemark, Norway. Ecol. Indic..

[6] Martín-López B., Iniesta-Arandia I., García-Llorente M., Palomo I., Casado-Arzuaga I., Amo D.G.D., Gómez-Baggethun E., Oteros-Rozas E., Palacios-Agundez I., Willaarts B. (2012). Uncovering Ecosystem Service Bundles through Social Preferences. PLoS ONE.

[7] Goldenberg R., Kalantari Z., Cvetkovic V., Mörtberg U., Deal B., Destouni G. (2017). Distinction, Quantification and Mapping of Potential and Realized Supply-Demand of Flow-Dependent Ecosystem Services. Sci. Total Environ..

[8] Wenke R. (1982). Review of Heartland of Cities: Surveys of Ancient Settlement and Land Use on the Central Floodplain of the Euphrates. Am. Anthropol..

[9] Núñez D., Nahuelhual L., Oyarzún C. (2006). Forests and Water: The Value of Native Temperate Forests in Supplying Water for Human Consumption. Ecol. Econ..

[10] Palacios-Agundez I., Onaindia M., Barraqueta P., Madariaga I. (2015). Provisioning Ecosystem Services Supply and Demand: The Role of Landscape Management to Reinforce Supply and Promote Synergies with Other Ecosystem Services. Land Use Policy.

[11] Uthes S., Matzdorf B. (2016). Budgeting for Government-Financed PES: Does Ecosystem Service Demand Equal Ecosystem Service Supply?. Ecosyst. Serv..

[12] Bukvareva E., Zamolodchikov D., Kraev G., Grunewald K., Narykov A. (2017). Supplied, Demanded and Consumed Ecosystem Services: Prospects for National Assessment in Russia. Ecol. Indic..

[13] Wolff S., Schulp C.J.E., Kastner T., Verburg P.H. (2017). Quantifying Spatial Variation in Ecosystem Services Demand: A Global Mapping Approach. Ecol. Econ..

[14] Bryan B.A., Ye Y., Zhang J., Connor J.D. (2018). Land-Use Change Impacts on Ecosystem Services Value: Incorporating the Scarcity Effects of Supply and Demand Dynamics. Ecosyst. Serv..

[15] Wickham J.D., Riitters K.H., Wade T.G., Vogt P. (2010). A National Assessment of Green Infrastructure and Change for the Conterminous United States Using Morphological Image Processing. Landsc. Urban Plan..

[16] Aparicio Uribe C.H., Bonilla Brenes R., Hack J. (2022). Potential of Retrofitted Urban Green Infrastructure to Reduce Runoff—A Model Implementation with Site-Specific Constraints at Neighborhood Scale. Urban For. Urban Green..

[17] Arthur N., Hack J. (2022). A Multiple Scale, Function, and Type Approach to Determine and Improve Green Infrastructure of Urban Watersheds. Urban For. Urban Green..

[18] Li K., Li C., Liu M., Hu Y., Wang H., Wu W. (2021). Multiscale Analysis of the Effects of Urban Green Infrastructure Landscape Patterns on PM2.5 Concentrations in an Area of Rapid Urbanization. J. Clean. Prod..

[19] Larondelle N., Lauf S. (2016). Balancing Demand and Supply of Multiple Urban Ecosystem Services on Different Spatial Scales. Ecosyst. Serv..

[20] Burkhard B., Kroll F., Nedkov S., Müller F. (2012). Mapping Ecosystem Service Supply, Demand and Budgets. Ecol. Indic..

[21] Gopalakrishnan V., Bakshi B.R., Ziv G. (2016). Assessing the Capacity of Local Ecosystems to Meet Industrial Demand for Ecosystem Services. AIChE J..

[22] Syrbe R.-U., Grunewald K. (2017). Ecosystem Service Supply and Demand—The Challenge to Balance Spatial Mismatches. Int. J. Biodivers. Sci. Ecosyst. Serv. Manag..

[23] van Oudenhoven A.P.E., Petz K., Alkemade R., Hein L., de Groot R.S. (2012). Framework for Systematic Indicator Selection to Assess Effects of Land Management on Ecosystem Services. Ecol. Indic..

[24] Villamagna A.M., Angermeier P.L., Bennett E.M. (2013). Capacity, Pressure, Demand, and Flow: A Conceptual Framework for Analyzing Ecosystem Service Provision and Delivery. Ecol. Complex..

[25] Baró F., Haase D., Gómez-Baggethun E., Frantzeskaki N. (2015). Mismatches between Ecosystem Services Supply and Demand in Urban Areas: A Quantitative Assessment in Five European Cities. Ecol. Indic..

[26] Shi Y., Shi D., Zhou L., Fang R. (2020). Identification of Ecosystem Services Supply and Demand Areas and Simulation of Ecosystem Service Flows in Shanghai. Ecol. Indic..

[27] Field C.B., Barros V., Stocker T.F., Dahe Q., Jon Dokken D., Ebi K.L., Mastrandrea M.D., Mach K.J., Plattner G.K., Allen S.K. (2012). Managing the Risks of Extreme Events and Disasters to Advance Climate Change Adaptation: Special Report of the Intergovernmental Panel on Climate Change.

[28] Field C.B., Barros V.R., Dokken D.J., Mach K.J., Mastrandrea M.D., Bilir T.E., Chatterjee M., Ebi K.L., Estrada Y.O., Genova R.C. (2014). Climate Change 2014 Impacts, Adaptation and Vulnerability: Part A: Global and Sectoral Aspects: Working Group II Contribution to the Fifth Assessment Report of the Intergovernmental Panel on Climate Change. Climate Change 2014 Impacts, Adaptation and Vulnerability.

[29] Chen B., Zhang H., Wang T., Zhang X. (2021). An Atmospheric Perspective on the Carbon Budgets of Terrestrial Ecosystems in China: Progress and Challenges. Sci. Bull..

[30] Misra A., Roorda M.J., MacLean H.L. (2013). An Integrated Modelling Approach to Estimate Urban Traffic Emissions. Atmos. Environ..

[31] Chiesura A. (2004). The Role of Urban Parks for the Sustainable City. Landsc. Urban Plan..

[32] Kessel A., Green J., Pinder R., Wilkinson P., Grundy C., Lachowycz K. (2009). Multidisciplinary Research in Public Health: A Case Study of Research on Access to Green Space. Public Health.

[33] Zhang Y., Liu Y., Zhang Y., Liu Y., Zhang G., Chen Y. (2018). On the Spatial Relationship between Ecosystem Services and Urbanization: A Case Study in Wuhan, China. Sci. Total Environ..

[34] Moran P.A.P. (1950). Notes on Continuous Stochastic Phenomena. Biometrika.

[35] Anselin L. (2010). Local Indicators of Spatial Association—LISA. Geogr. Anal..

[36] Meerow S., Newell J.P. (2017). Spatial Planning for Multifunctional Green Infrastructure: Growing Resilience in Detroit. Landsc. Urban Plan..

[37] Morse W.C., Stern M., Blahna D., Stein T. (2022). Recreation as a Transformative Experience: Synthesizing the Literature on Outdoor Recreation and Recreation Ecosystem Services into a Systems Framework. J. Outdoor Recreat. Tour..

[38] Gangahagedara R., Subasinghe S., Lankathilake M., Athukorala W., Gamage I. (2021). Ecosystem Services Research Trends: A Bibliometric Analysis from 2000–2020. Ecologies.

[39] Jeong D., Kim M., Song K., Lee J. (2021). Planning a Green Infrastructure Network to Integrate Potential Evacuation Routes and the Urban Green Space in a Coastal City: The Case Study of Haeundae District, Busan, South Korea. Sci. Total Environ..

